# Serum prolidase activity in benign joint hypermobility syndrome

**DOI:** 10.1186/1471-2474-15-75

**Published:** 2014-03-11

**Authors:** Serda Em, Demet Ucar, Pelin Oktayoglu, Mehtap Bozkurt, Mehmet Caglayan, Ismail Yıldız, Osman Evliyaoglu, Kemal Nas

**Affiliations:** 1 Department of Physical Medicine and Rehabilition, Division of Rheumatology, Dicle University Faculty of Medicine, Diyarbakir, Turkey; 2 Department of Biostatistics, Dicle University Faculty of Medicine, Diyarbakir, Turkey; 3 Department of Biochemistry, Dicle University Faculty of Medicine, Diyarbakir, Turkey

**Keywords:** Hypermobility, Prolidase, Ligament, New York rating test

## Abstract

**Background:**

Moderate joint laxity is widespread in many joints of the body, and this condition is considered to be caused by an abnormality in the collagen structure. This study was carried out to determine the serum prolidase activity in female patients with benign joint hypermobility syndrome (BJHS), and to evaluate its correlation with their clinical features.

**Methods:**

A total of 45 patients with BJHS and 40 healthy controls were included in the study. All of the patients with BJHS met the Beighton diagnostic criteria. All the patients and the control group underwent a comprehensive examination of the locomotor system and took the New York Posture Rating Test. The examination and test results were recorded. Serum prolidase activity was measured in both the groups.

**Results:**

Prolidase activity was significantly lower in patients with BJHS (479.52 ± 126.50) compared to the healthy controls (555.97 ± 128.77) (p = 0.007). We found no correlation between serum prolidase activity and Beighton scores or New York rating test scores. On the other hand, mean prolidase activity was significantly lower in patients with pes planus or hyperlordosis compared to those without (p = 0.05, p = 0.03, respectively). We did not find such a correlation with the other clinical features.

**Conclusions:**

Significantly lower prolidase activity in patients with BJHS suggests that prolidase may affect the collagen metabolism and cause hyperlaxity.

## Background

Benign joint hypermobility syndrome (BJHS) is a hereditary disease characterised by musculoskeletal symptoms in patients with widespread joint laxity, independent of a systemic rheumatoid disease [1]. The frequency of BJHS varies between 5–57% in young females and 2–35% in males [2]. Besides, the incidence of BJHS is higher in females compared to males and decreases with age [3]. It may develop with some musculoskeletal symptoms which are affiliated with bones, tendons, muscles, ligaments, joints and spine. Impaired stability in the joint causes pathologies in the joint because of insufficient support of the collagen connective tissue in the nonjoint [4,5].

Collagen is an abundant protein of the body. It is a major component of skin, tendons, ligaments, joint capsules and blood vessels [6]. Ligaments are dense bands of articular tissues that connect bones together. Those found in the knee are hypocellular ligaments and are composed of type I and III collagens, proteoglycans, elastin and water [7]. Maximum possible range of motion is determined by the tension of ligaments, hence by their motion restricting features. Therefore, the primary underlying cause of the hypermobility is the ligament laxity [8,9]. Reduced thickness of collagen fibrils in patients with BJHS, as suggested by the electron microscopic examination of the skin biopsy, supports this view [10].

Prolidase is an important enzyme that takes part in the collagen formation and degradation [11,12] and promotes recycling of the residues of proline, acquired from collagen degradation [13]. Prolidase deficiency affects the recycling of residues of proline in the collagen re-synthesis [14,15], consistent with the findings by some earlier studies that have reported increased prolidase activity in the fibrotic process of the liver [16], including a work in rats [17].

A study conducted in children with hypermobility reports a lower prolidase activity compared with that in control [18] although conducting a similar study is not seen in adult patients with BJHS. The presenst study involves female adult patients with BJHS. We speculate that defects in the collagen structure might be responsible for ligament laxity in patients with BJHS. Therefore we assume that prolidase deficiency might lead to a reduction in the proline content of the collagen and change the collagen structure. This study aims to evaluate the existing clinical features in patients with BJHS and compare the prolidase activity in the patients with that in the healthy controls and also investigate the correlation between prolidase and clinical features.

## Methods

### Study population and assessments

A total of 45 female patients of reproductive age diagnosed with joint hypermobility and 40 healthy controls were included in the study. Ethical board’s approval was obtained, patients and healthy controls were fully informed about the study, and their written consent was taken before the study was performed. Exclusion criteria were as follows: pregnancy, breastfeeding, use of oral contraceptives, menstrual irregularity and existence of any neurological, rheumatoid, skeletal, metabolic or collagen disease. Medical histories of the participants were taken. Furthermore, the participants underwent physical examinations and they were checked for routine hematologic and biochemical parameters. Physical examinations and assessments were conducted by the same team of physiatrist early in the morning, at the room temperature, with patients wearing only underwear and no shoes.

Beighton scores were used in an effort to diagnose patients with BJHS (Table 1) [19]. The lowest score was 0 and the highest score was 9. The scores at or above 4 were diagnosed as BJHS. The patients and healthy controls were assessed for the existence of joint pain, widespread pain throughout the body, carpal tunnel syndrome, joint subluxation, hyperkyphosis, hyperlordosis and pes planus. New York Posture rating test was used to assess the posture [20]. In this assessment, the patients were scored from the front, back, and sideways by observing their possible posture changes in 13 different parts of body. The participant was scored 5 if she had a straight posture, 3 if she had a moderately impaired posture and 1 if she had a severely impaired posture. The minimum total sum was 13 and maximum total sum was 65 when 13 parts were scored.

**Table 1:** Nine-point Beighton hypermobility score

**Task**	**Right**		**Left**
1. Passively dorsiflex the fifth metacarpophalangeal joint to ≥ 90°	1		1
2. Oppose the thumb to the volar aspect of the ipsilateral forearm	1		1
3. Hyperextend the elbow to ≥ 10°	1		1
4. Hyperextend the knee to ≥ 10°	1		1
5. Place hands flat on the floor without bending the knee		1	
Total possible score		9	

### Blood sample

Following the examination of locomotor system, the Beighton manoeuvres and the New York posture analyses, blood samples were collected from 45 consecutive females with joint hypermobility, using a cut-of value of 4 or more, and 40 healty controls at 9:00–11:00 a.m. Serum was isolated from the blood after it was kept at room temperature for 30 minutes and centrifuged at 3000 rpm for 15 minutes. Serum samples were coded and kept at -40°C for further spectrophotometric analyses.

### Determination of prolidase

Activity of plasma prolidase (U/L) were determined by a spectrophotometric method that measures the proline levels produced by prolidase. The supernatant was diluted up to two folds by normal saline. Twenty-five microliters of mixture was preincubated with 75 μL preincubation solution (50 mmol/L Tris HCl buffer pH 7.0 containing 1 mmol/L glutathione and 50 mmol/L MnCl2) at 37°C for 30 minutes. The reaction mixture containing 144 mmol/L gly–pro, pH 7.8 (100 μL), was incubated with 100 μL preincubated sample at 37°C for 5 minutes. 1 mL glacial acetic acid was added to the mixture in order to cease the incubation reaction. After adding 300 μL Tris HCl buffer, pH 7.8, and 1 mL ninhydrin solution (3 g/dL ninhydrin was melted in 0.5 mol/L orthophosphoric acid), the mixture was incubated at 90°C for 20 minutes and then cooled with ice. Later on, absorbance was measured at a 515 nm wavelength to determine the proline by using the method recommended by Myara et al. [16,18]. This method is a modified version of Chinard’s method [21]. The intra-assay and interassay coefficients of variation (CVs) were both lower than 7%.

### Statistical analysis

Measurement variables were expressed in mean ± standard deviation, while categorical variables were presented in numbers and percentages (%). Kolmogorow-Smirnow test was used to analyse the compliance of datasets with the normal distribution. Student-t test was used to compare the mean values of the group that displayed a normal distribution, and Mann–Whitney U test was used for the group that did not. Furthermore, Spearman’s correlation test was used to assess the correlation between serum prolidase activity and Beighton scores, New York rating test scores, joint pain, myalgia, carpal tunnel syndrome, shoulder impingement, joint subluxation, hyperkyphosis, hyperlordosis and pes planus. Varying frequencies among the categorical groups were evaluated by Chi-square test. Fisher’s exact test was used when the expected values were lower than 5. A p value below 0.05 was regarded to be statistically significant. All the analyses were performed by Statistical Package for Social Sciences software version 15.0 for Windows.

### Power analysis

In this specific study, the sample size of patients and controls were determined as at least 40 according to α = 0.05 and β = 0.15. Mean serum prolidase activity were determined in the patients and controls (479.52 ± 126.50, 555.97 ± 128.77 respectively). The power of statistical analysis for study population was 0.87 according to the given effect size (population means of 479.52 vs 555.97), SD (126.50 vs 128.77), sample sizes (45 and 40), and alpha (0.050, two-tailed).

## Results

We found no statistically significant difference between the two groups in terms of age, weight, height and menstrual status (p > 0.05). Mean Beighton score was significantly higher in the patients (min: 4, max: 9; mean: 6.64 ± 1.28). Mean serum prolidase activity was significantly lower in the patients (479.52 ± 126.50 pg/mL) compared to the control group (555.97 ± 128.77 pg/mL) (p = 0.007). New York Posture rating test scores were significantly lower in patients with BJHS (49.0 ± 16.2) compared to the control group (58.8 ± 3.5) (p = 0.001) (Table 2).

**Table 2:** Prolidase activity and demographic characteristics of the study groups (mean ± SD)

	**Hypermobile (n = 45)**	**Normal (n = 40)**	**p**
Age (years)	25.26 ± 6.69	25.45 ± 7.14	0.9
Weight (kg)	55.63 ± 8.91	55.73 ± 8.13	0.9
Height (cm)	161.6 ± 5.01	161.32 ± 4.15	0.9
BMI (kg/m^2^)	21.32 ± 3.55	21.41 ± 3.06	0.5
Beighton score (mean ± SD)	6.64 ± 1.28	0.65 ± 0.89	<0.001
Min	4	0	
Max	9	3	
Prolidase (pg/mL)	479.52 ± 126.50	555.97 ± 128.77	0.007
New York posture rating test score	49.04 ± 16.22	58.82 ± 3.56	0.001

BMI, Body mass index.

Significance was defined as p < 0.05.

Clinical features of the patients and the controls are shown in Table 3. The frequency of joint pain (p = 0.000), myalgia (p = 0.01), shoulder impingement (p = 0.05), pes planus (p = 0.01) and hyperkyphosis was significantly higher in patients with BJHS compared to the control group. No significant difference was found between the groups with regard to the frequency of other clinical parameters (p > 0.05) (Table 3). Serum prolidase activity was significantly lower in patients with BJHS having pes planus compared to those without pes planus (p = 0.05). Furthermore, serum prolidase activity was significantly lower in patients with BJHS having hyperlordosis compared to those without hyperlordosis (p = 0.03) Table 4. No significant correlation was found between the serum prolidase activity and age, BMI, Beighton scores and New York posture test scores.

**Table 3:** The clinical features of study groups

**Features**	**Hypermobility n = 45 (%)**	**Normal n = 40 (%)**	**p**
Arthralgia	15 (33.3)	10 (25)	<0.001
Myalgia	25 (55.6)	11 (27.5)	0.01
Carpal tunnel syndrome	4 (8.9)	2 (5)	0.11
Impingement syndrome	5 (11.1)	3 (7.5)	0.05
Subluxation	3 (6.7)	0 (0)	0.24
Hyperlordosis	30 (66.7)	28 (70)	0.818
Pes planus	26 (57.8)	12 (30)	0.01
Hyperkyphosis	28 (62.2)	9 (22.5)	<0.001

Significance was defined as p < 0.05.

**Table 4:** The activity of prolidase associated with clinical symptoms and findings

	**Prolidase, (mean ± SD)**		**p**
	**Presence**	**Absence**	
Arthralgia	452.34 ± 121.78	513.51 ± 127.04	0.10
Myalgia	452.87 ± 124.96	509.98 ± 124.18	0.13
Carpal tunnel syndrome	483.36 ± 136.82	479.15 ± 127.27	0.95
İmpingement syndrome	468.72 ± 116.56	480.87 ± 129.01	0.84
Subluxation	455.70 ± 45.10	481.23 ± 130.50	0.74
Hyperlordosis	422.66 ± 121.60	507.96 ± 120.76	0.03
Pes planus	437.23 ± 116.09	510.43 ± 126.92	0.05
Hyperkyphosis	477.34 ± 132.40	483.11 ± 120.02	0.88

Significance was defined as p < 0.05.

## Discussion

Collagen is the main component of ligament structure. Prolidase is an enzyme which can influence the collagen structure in patients with BJHS. There is a study in the literature which evaluates the prolidase activity in children with hypermobility [18]. The current work is the first study to investigate the prolidase activity in adult females. The study is different from the others in two points; it has an adult target group and it establishes the correlation between prolidase and clinical features.

In this study, we found that mean serum prolidase activity was significantly lower in patients with BJHS compared to the healthy controls. Besides, the frequency of joint pain, myalgia, shoulder impingement, hyperkyphosis and pes planus was significantly higher in patients with BJHS. Serum prolidase activity was significantly lower in patients with pes planus and hyperlordosis. On the other hand, we found no significant correlation between prolidase and Beighton scores or New York posture test scores.

Benign hypermobility is a clinical syndrome characterised by greater than normal active or passive range of motion in joints, independent of a systemic rheumatoid disease. Although various enzyme and hormone studies have been conducted in this field [22,23], no full light has been shed on the etiopathogenesis of BJHS. Ligaments are hypocellular and consist of collagen, proteoglycans, elastin and water [7]. Such structural features as stiffness and flexibility of the ligament play an important role in the creation of normal joint motions. Therefore, changes in the collagen structure and the resulting ligament laxity are considered to be highly responsible for hypermobility.

Prolidase enzyme plays an important role in the regulation of collagen metabolism. Glisinprolin, a substrate of prolidase, exits in the collagen structure [24]. There are some reports stating that prolidase activity is significantly different in patients with variety of diseases where it is thought that their pathogenesis involves the pathology of collagen biosynthesis [11,25]. Significantly higher activity of prolidase is reported in patients with idiopathic clubfoot [25]. Previously, it has been found that prolidase activity is inhibited by collagen degradation products [21]. In a study, Galicka et al. [21] found lower prolidase enzyme activity in osteogenesis imperfecta cells. In another study conducted on children with joint hypermobility, who met the Beighton diagnostic criteria, Yazgan et al. [18] found lower prolidase activity in the patients compared to the controls, which was not statistically significant. Both studies concluded that decreased prolidase activity might affect the cellular growth and the collagen metabolism. Similarly, we found significantly lower prolidase activity in patients with BJHS, suggesting that prolidase might affect the collagen metabolism, partially resulting in changes in the ligament structure and paving the way for hypermobility. However, our study did not show any correlation between prolidase enzyme activity and Beighton scores. This may be attributed to the fact that the average value of scores (mean ± SD: 6.64 ± 1.28; median: 6.00) is close to the lower limit value and/or the 50% of patients have 5 and 6 scoring values.

The number of studies that have evaluated the joint and non-joint symptoms in patients with BJHS is rather limited. In their study, Mishra et al. found that 31% of the patients with BJHS had joint pain, while 10% had ligament injury, another 10% had tendinopathy and 4% had subluxation [26]. Furthermore, Shiari et al. [27] found that the frequency of joint pain was % 35.4 among patients with BJHS, whereas Yazgan et al. concluded that 30% of the children with BJHS had joint pain, 10% had pes planus and 4% had myalgia [18].

A study which investigated the postures of patients with BJHS found significant differences between the patients with BJHS and the control group with regard to posture scores and pain [28]. All the clinical signs and symptoms evaluated under this study were significantly higher and New York posture rating test scores were significantly lower in patients with BJHS. Besides, such clinical features as joint pain, myalgia, shoulder impingement and hyperkyphosis had a significant correlation with the joint laxity in these patients. Poor posture is significant in the management of BJHS, and its long term effects must be taken into account when planning a treatment for such patients. Reviewing the literature, we have not seen any study which evaluates the frequency of carpal tunnel syndrome, hyperkyphosis and hyperlordosis in patients with BJHS. Our findings support the literature with reference to the joint pain and tendinopathy. On the other hand, we found lower values than those in the literature, particularly with regard to the values found for myalgia, pes planus and subluxation. This might be attributed to the genetic variations and environmental factors.

Being a cross sectional study is one of the restrictive factors in our study. Another restrictive factor is that our study did not involve any male patients. Given the fact that hypermobility is more prevalent among females, we could not find enough number of male patients and excluded this group from the study. Not considering the possible factors affecting prolidase activity may be another limitation of the present study.

## Conclusions

Prolidase is an important enzyme that takes part in the collagen formation and degradation. It is particularly influential in the last stage where the imminopeptidases which contain C-terminal proline and hyroxyproline split. We performed this study with assumption of a possible correlation between collagen defects and serum prolidase activity in patients with BJHS. We found that serum prolidase activity was significantly lower in the patients compared to the healthy controls. Therefore, we suggest that the lower serum prolidase activity may be an important etiologic factor in the etiopathogenesis of BJHS. However, in order to attain further knowledge about the role of prolidase in joint and the process of the disease, more comprehensive studies on a wider range of population are required.

## Competing interests

The authors’ declare that they have no competing interest.

## Authors’ contributions

SE was the main investigator and involved in the study design, data collection, analysis and drafting of the manuscript. DU was involved in the study design, data analysis, and providing the final draft. PO contributed to the idea, study design, data analysis and, drafting of the manuscript. MB was involved in the study design, and data analysis. MC was participated in the data analysis and, drafting of the manuscript. IY data contributed to analysis and interpretation. OE involved in the study design, and data analysis. KN was participated critical revision of the article. All authors read and approved the final manuscript.

## Pre-publication history

The pre-publication history for this paper can be accessed here:

http://www.biomedcentral.com/1471-2474/15/75/prepub
